# 4398 A Computational Psychiatry Approach to Addiction Using Neuroeconomics Translated Across Species

**DOI:** 10.1017/cts.2020.365

**Published:** 2020-07-29

**Authors:** Brian Sweis, Jazmin Camchong, Samantha Abram, Ann Haynos, Sheila Specker, Kelvin Lim, Angus MacDonald, Mark Thomas, David Redish

**Affiliations:** 1 University of Minnesota CTSI

## Abstract

OBJECTIVES/GOALS: Decision-making impairments in addiction can arise from dysfunction in distinct neural circuits. Such processes can be dissociated by measuring complex, computationally distinct behaviors within an economic framework. We aim to characterize computational changes conserved across models of addiction. METHODS/STUDY POPULATION: We used neuroeconomic tasks capable of dissociating neurally separable decision processes using behavioral analyses equally applicable to humans and rodents. We tested 12 human cocaine-users and 9 healthy controls on the Web-Surf task designed to match the rodent Restaurant Row task on which 27 mice were trained and then exposed to saline (n = 10), cocaine (n = 7), or morphine (n = 10). All subjects foraged for rewards (humans: entertaining videos; mice: food) of varying costs (1-30s delays) and subjective value (humans: genres; mice: flavors) by making serial accept or reject decisions while on a limited time budget, balancing the utility of wanting desirable rewards despite conflicting costs. RESULTS/ANTICIPATED RESULTS: When encountering unique offers for rewards with a delay above one’s willingness to wait, cocaine-treated mice like cocaine-exposed humans were less likely to appropriately reject economically disadvantageous offers. Furthermore, these mice and humans did so despite spending more time deliberating between future options. In contrast, morphine-treated mice displayed distinct impairments when given the opportunity to correct past mistakes, a process we previously demonstrated was uniquely sensitive to alterations in strength of synaptic connectivity of the infralimbic-accumbens shell circuit in mice. We anticipate human opioid-users will mirror these latter, computationally distinct findings. DISCUSSION/SIGNIFICANCE OF IMPACT: These data elucidate facets of addiction shared across species yet fundamentally distinct between disease subtypes. Our translational approach can help shed light on conserved pathophysiological mechanisms in order to identify novel diagnostic parameters and computational targets for intervention.

